# Unreported VOC Emissions from Road Transport Including from Electric Vehicles

**DOI:** 10.1021/acs.est.3c00845

**Published:** 2023-05-16

**Authors:** Samuel J. Cliff, Alastair C. Lewis, Marvin D. Shaw, James D. Lee, Michael Flynn, Stephen J. Andrews, James R. Hopkins, Ruth M. Purvis, Amber M. Yeoman

**Affiliations:** † Wolfson Atmospheric Chemistry Laboratories, University of York, York YO10 5DD, United Kingdom; ‡ National Centre for Atmospheric Science, University of York, York YO10 5DD, United Kingdom; § School of Earth and Environmental Sciences, University of Manchester, Manchester M13 9PL, United Kingdom

**Keywords:** air pollution, ethanol, screenwash, volatile chemical products, urban atmosphere

## Abstract

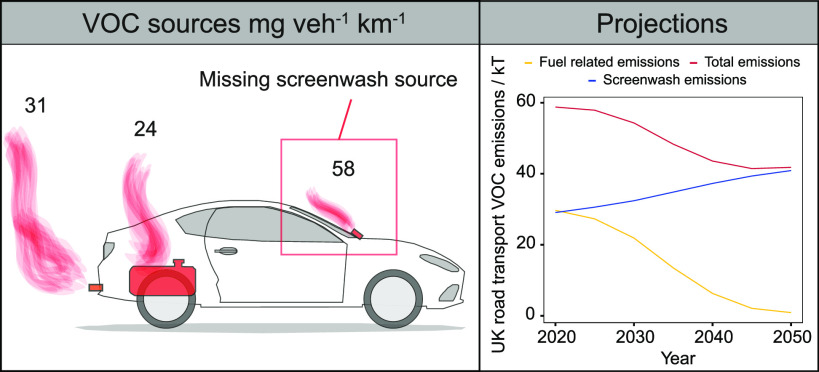

There are widespread policy assumptions that the phase-out of gasoline and diesel internal combustion engines will over time lead to much reduced emissions of Volatile Organic Compounds (VOCs) from road transport and related fuels. However, the use of real-world emissions measurements from a new mobile air quality monitoring station demonstrated a large underestimation of alcohol-based species in road transport emissions inventories. Scaling of industry sales statistics enabled the discrepancy to be attributed to the use of ancillary solvent products such as screenwash and deicer which are not included in internationally applied vehicle emission methodologies. A fleet average nonfuel nonexhaust VOC emission factor of 58 ± 39 mg veh^–1^ km^–1^ was calculated for the missing source, which is greater than the total of all VOCs emitted from vehicle exhausts and their associated evaporative fuel losses. These emissions are independent of the vehicle energy/propulsion system and therefore applicable to all road vehicle types including those with battery-electric powertrains. In contrast to predictions, vehicle VOC emissions may actually increase given a predicted growth in total vehicle kilometers driven in a future electrified fleet and will undergo a complete VOC respeciation due to the source change.

## Abstract 2

Measurements show that screenwash application dominates vehicular VOC emissions and is missing from road transport inventories.

## Introduction

Road traffic has long been an important source of air pollution to urban environments both directly, and indirectly, contributing to five classes of major air pollutants (NO_*x*_, particulate matter (PM), O_3_, VOCs, and CO). However, with increasingly stringent emissions legislation and continued uptake of air pollution abatement strategies, the dominant sources are changing.^1^ A wealth of research has been dedicated to understanding nonexhaust PM (for example, brake and tire wear), since these are sources that will be present despite fleet electrification.^2^ However, very little consideration has been given to nonexhaust VOC emissions.

There have been large decreases in both emissions and some concentrations of VOCs since the mid-1990s in Europe and North America following the universal implementation of the three-way catalytic converter (exhaust control) and the carbon canister (evaporative control).^3^ Light duty vehicle regulated emissions standards for NMHC + NO_*x*_ have decreased by 97% in the U.S. (Tier 1–3) and by 80–85% in the EU (Euro 1–6),^4^ with further improvement planned.

VOC emissions are unusual compared to other gaseous air pollutants because they are a summed group of thousands of different compounds rather than a single chemical. Different VOC species play different roles in atmospheric chemistry depending on their reactivity and functionality. For example, short chain alkenes have a low secondary organic aerosol formation potential, but a high ozone formation potential resulting from their high reactivity with the hydroxyl radical. Aromatic species exhibit different properties being both precursors to ozone and particulate matter.^5^ To fully understand atmospheric and potential health implications of VOCs, it is not sufficient to solely monitor the change in total VOC burden to the atmosphere, but also to accurately determine the change in composition.

**Figure 1:**
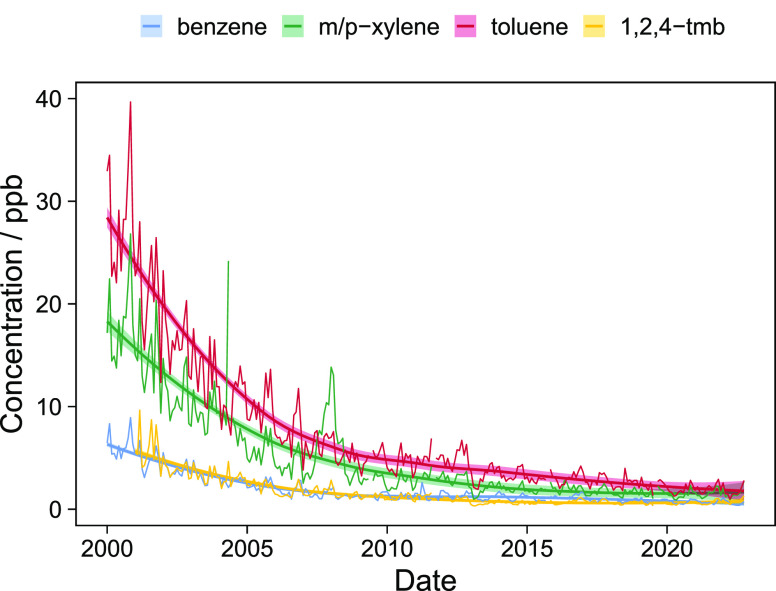
Time series benzene, *m*/*p*-xylene, toluene, and trimethylbenzene at the Marylebone road, central London air quality monitoring station.

Typically, only a small number of VOC species are monitored routinely, and many oxygenated VOCs are not measured at all despite comprising an increasing fraction of emissions.^1^ In the U.K. only 4 of the 10 most abundant VOCs are now being measured by national air quality monitoring networks.^6^ Current observations in Europe focus on those VOCs that are distinctive of fossil fuels and combustion and have convincedly tracked the downward trends in concentrations related to gasoline vehicles (Figure 1). Policy projections for the future of VOC emissions in high income countries show downward trends in VOC emissions as older vehicles leave the fleet to be replaced in the medium to long-term by electric vehicles. It appears intuitive that vehicles without fuels and combustion will be VOC-free in terms of their operating emissions.

The United Kingdom has a detailed National Atmospheric Emissions Inventory (NAEI) for VOCs which is constructed bottom up, often using industry supplied sales statistics for solvent containing products. It is speciated into more than 600 different individual VOCs. A curious observation from that inventory is that there are industry reported classes of solvent-containing products, labeled as “car care”, that appear to give rise to a larger mass of emissions than that from fuel evaporation and tailpipe exhaust.^7^ A large fraction of this is thought to be the application of screenwash which contains VOCs in the form of alcohol content for their antifreeze properties. Screenwash has in the past received some attention and consideration, in particular in the U.S., as an important source of air pollution. California, for example, limits their summer formula to 1% VOC content with only certain areas allowed to sell winter blends up to 25% VOC content.^8^ This group of products, at least in the U.K. NAEI, appears to contribute 6% of all U.K. VOC emissions. However, only a very small number of countries, namely The Netherlands and parts of Scandinavia, produce inventories with this degree of speciation and product granularity.^9^ Moreover, the presence of this apparently large VOC source has never been experimentally verified in the field. Here we utilize a new mobile measurement platform to calculate real-world road transport VOC emission factors via a roadside increment-type analysis in Manchester, U.K. This methodology has previously been used for studies of nonexhaust particulate matter emissions,^10^ and accommodated the quantification of nonfuel related VOC emissions at the roadside. We compare measurements to inventory estimated emissions and outline the potential implications of the findings on future emissions scenarios, policy, and atmospheric chemistry.

## Materials and Methods

### Measurement Locations

The locations of the two measurement sites are shown in Figure S1 of the Supporting Information (SI). The roadside site was situated on Upper Brook Street (53° 27′59.9′′N, 2° 13′44.9′′W), which is a key arterial road for transport into and out of Central Manchester and at the location of the roadside site consisted of four lanes. The Manchester Air Quality Supersite (MAQS) is located at the Firs Botanical Gardens (53° 26′38.9′′N, 2° 12′51.1′′W) on the University of Manchester Fallowfield Campus, and is representative of urban background air. Two, three-week measurement periods took place during the Observation System for Clean Air project (OSCA) in July 2021 and February 2022.

### Instrumentation

The WACL Air Sampling Platform (WASP) was deployed as the roadside measurement site. The WASP has been previously described in detail,^11^ with an updated suite of instrumentation presented in Figure S1. The Manchester Air Quality Supersite (MAQS) used as the urban background site carries out long-term measurements of gases, aerosols, and meteorology.^12−14^ The instrumentation used to measure each atmospheric species is described below.

#### VOCs

At the roadside, a Voice 200 Ultra Selected-Ion Flow Tube Mass Spectrometer (SIFT-MS) (Syft Technologies Ltd., New Zealand) was used to quantify VOC mixing ratios. The theory of operation is described in detail elsewhere in the literature,^15^ with the instrument being operated using a flow tube pressure of 460 mTorr. Sampling from an in-house built palladium alumina-based zero air generator assessed the instrument background for 5 min of each hour. The 5 min average background mixing ratio was subtracted from the ambient mixing ratio measurements of the corresponding hour. Sensitivities for the compounds detected by SIFT-MS were determined every 3 days from automated multipoint calibrations performed using an in-house developed dilution unit. This used a 1 ppm gravimetrically prepared standard of different VOCs in ultrahigh purity nitrogen (National Physics Laboratory, U.K.) diluted with ambient humidity zero air. At the background MAQS, VOCs were measured using Thermal Desorption-Gas Chromatography coupled with Flame Ionization Detection (TD-GC-FID) (Agilent Technologies Inc., U.S.A.). Calibration gas was provided from a working standard cylinder comprising a sample of VOCs (material number: 177664-AL-HC, BOC Special Gases) diluted to 1.2 ppb per component in purified nitrogen (cylinder number: D035781, Air Liquide S.A., France), linked to an NPL30 primary calibration standard (National Physical Laboratory, UK). Further details on the VOC measurements are provided in the SI.

#### Nitrogen Oxides

NO_*x*_ (NO + NO_2_) was measured at the roadside using the Iterative CAvity enhanced Differential optical absorption spectroscopy system (ICAD) (Enviro Technology Services Ltd., U.K.).^16^ Urban background NO_*x*_ was calculated via the sum of two separate measurements of NO and NO_2_. NO was measured using a Thermo 42i- (Thermo Fisher Scientific Inc., U.S.A.), and NO_2_ using a T500U Cavity Attenuated Phase Shift (CAPS) analyzer (Teledyne API., U.S.A.).

#### Carbon Dioxide

An Ultraportable Greenhouse Gas Analyzer (UGGA) (Los Gatos Research Inc., U.S.A.) was used to quantify mixing ratios of CH_4_, CO_2_, and H_2_O (1 Hz data acquisition) at the roadside. The instrument utilizes Off-Axis Integrated-Cavity Output Spectroscopy (OA-ICOS) to directly quantify mixing ratios of the three species.^17,18^ The instrument was linearly calibrated using a three-point calibration curve, using standards traceable to the WMO scale. CO_2_ was measured at the supersite using a Multigas Carbon Emissions Analyzer (MGCEA) (Los Gatos Research Inc., USA), capable of simultaneous measurements of CO_2_, CH_4_, CO, and H_2_O. The MGCEA operates using the same measurement principles as the UGGA.

#### Traffic Data

To gather insight into the type and number of vehicles traveling by the measurement site, traffic counts, vehicle type, and hourly average speed data were gathered by a Vivacity traffic camera. The traffic sensor uses machine learning algorithms to enable accurate detection, classification, and analysis of different transport modes and traffic movement. Vehicle type was broken down into the following categories: buses and coaches, cars and vans, cars with a trailer, Heavy Goods Vehicles (HGVs), motorcycles and rigids. The camera is owned by Transport for Greater Manchester (TfGM), and the data was provided by Manchester-i, a data solution that collects, hosts, and exposes city open data to a broad set of researchers and end-users operating/interested in urban-related disciplines. Across the two periods, emissions from a total of 754 519 vehicles were measured.

### Emission Factor Calculation

#### Real World Emission Factors

Fleet average emission factors for different VOC species were calculated from hourly speed-dependent emission factors of a tracer species, the incremental concentrations of the tracer species and VOCs at the roadside in comparison with an urban background site, and an assumption that the emitted tracer and VOC species were transported and diluted in the same way in the atmosphere, as has been done previously in the literature.^10,19,20^ In this study, both CO_2_ and NO_*x*_ were used as tracers. The following three steps show how this was done.

(1) The roadside increment of a tracer species (Δ*C*_*T*_) with well-known emission factors was calculated. This was done using eq 1, where the roadside concentration (*C*_TRoadside_) was measured by the WASP, and the background (*C*_TBackground_) was measured at the MAQS urban background location.
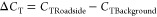
1
(2) Hourly emission factors for the tracer species were calculated and combined with the roadside increment in part (a) to calculate a correction factor for the dilution of the emissions between the two sites. Hourly tracer species speed-dependent emission factors (EF_T_) were obtained from the Department for Environment, Food, and Rural Affairs (Defra) Emission Factor Toolkit (V11.0).^21^ Due to the nearby location of the TfGM Vivacity traffic camera, an hourly detailed breakdown of traffic counts (*n*) and type (i) was able to be used as an input, along with hourly average speed, the Urban (not London) road-type setting and the appropriate year of measurement (2021 or 2022 depending on the measurement campaign period). The hourly dilution correction (dT) was then calculated from eq 2 using the tracer emission factor for each vehicle type and the dry roadside increment concentration.
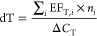
2
3) The fleet average emission factor for a species *x* (EF_fleet,*x*_), was then calculated from the roadside increment of that species (Δ*C*_*x*_), the dilution factor of the tracer species and the total number of vehicles on the road during the hour (*n*_tot_) in eq 3.
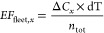
3

### Inventory Estimated Emission Factors

Inventory estimated emissions were calculated using the international reference methods of COPERT (Calculation Of air Pollutant Emissions from Road Transport) following UK-specific guidance presented in the “Methodology for the UK’s Road Transport Emissions Inventory” report.^22^ The U.K. road transport emissions inventory in turn follows the methodology outlined in the EMEP/EEA “Air Pollutant Emission Inventory Guidebook”,^23^ which uses emission factors in COPERT. A full description of the data used for the COPERT calculations is given in the SI.

## Results and Discussion

### Roadside vs Background Atmospheric Concentrations

**Figure 2:**
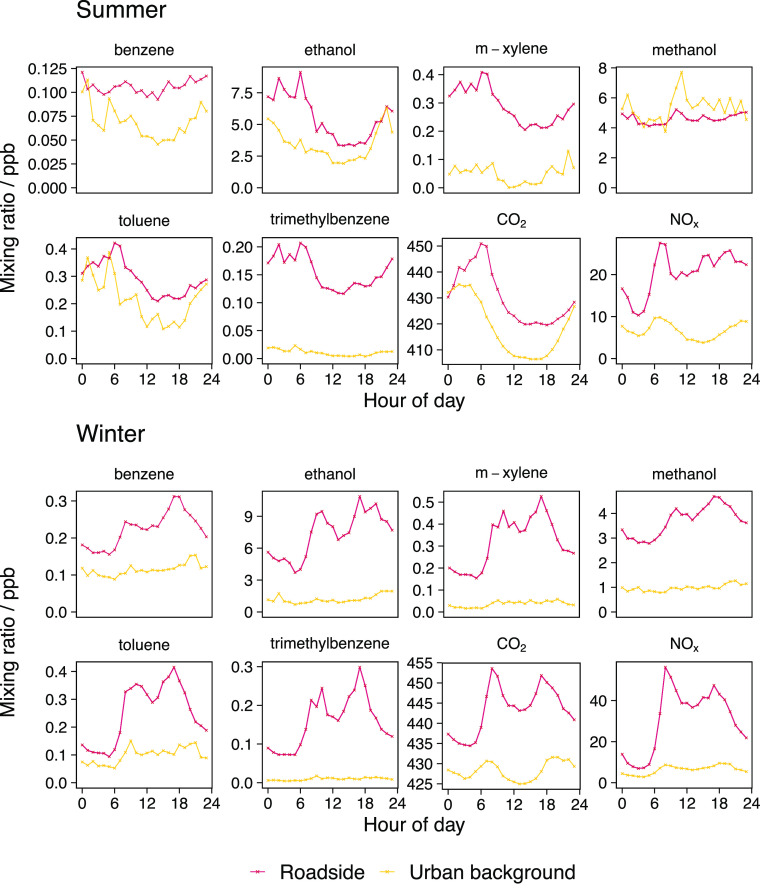
Median average diurnal profiles for six VOC species (benzene, ethanol, *m*-xylene, methanol, toluene, and trimethylbenzene) and both tracer species (CO_2_ and NO_*x*_) for the summer and winter at both measurement sites.

**Figure 3:**
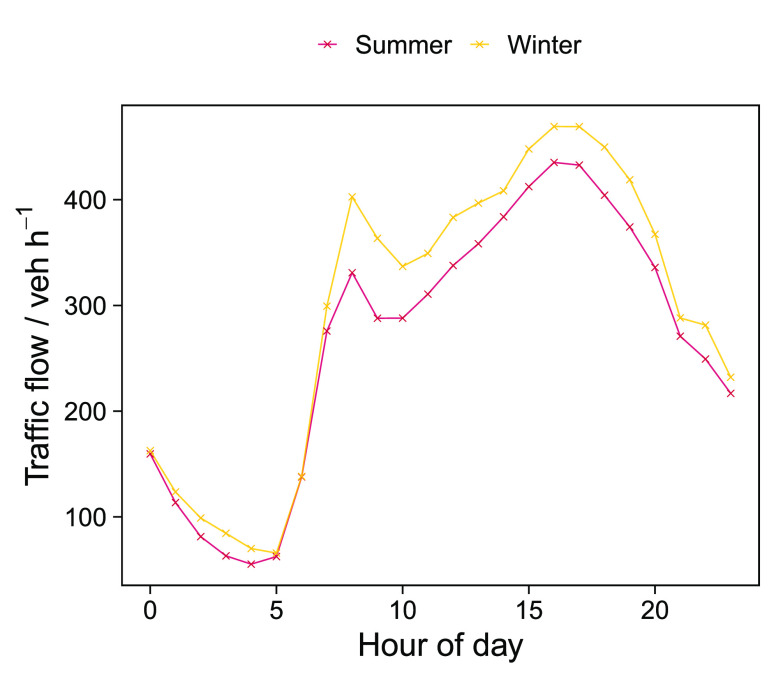
Median average diurnal profiles for traffic counts at the roadside site during the summer and winter measurement periods.

Ethanol and methanol were consistently the most abundant VOCs measured at both the roadside and the urban background site (Figure 2, see Table S1 for the full list of VOC species measured at the roadside). Throughout the day, concentrations an order of magnitude higher than the aromatic species were observed as is in agreement with previous measurements made in London, U.K.^24^ All species showed a positive roadside increment apart from methanol in the summer; methanol at the urban background site was greater than at the roadside due to the influence of biogenic emissions from the botanical gardens where the MAQS is located in. There was a notable contrast in diurnal shape between the seasons due to the impact of meteorology in the summer. Summer diurnals were driven by boundary layer height where a decrease in concentration after the morning rush hour is observed in line with increasing boundary layer height, before rising again in the evening as the boundary layer height begins to fall. Roadside winter diurnal profiles for all VOCs presented consistently tracked traffic flow (Figure 3), and the tracer species (NO_*x*_ and CO_2_), with a peak in the morning and evening in line with rush hour increases in traffic. The urban background concentrations remained low and stable with minimum influence from traffic.

### Emission Factors

**Figure 4:**
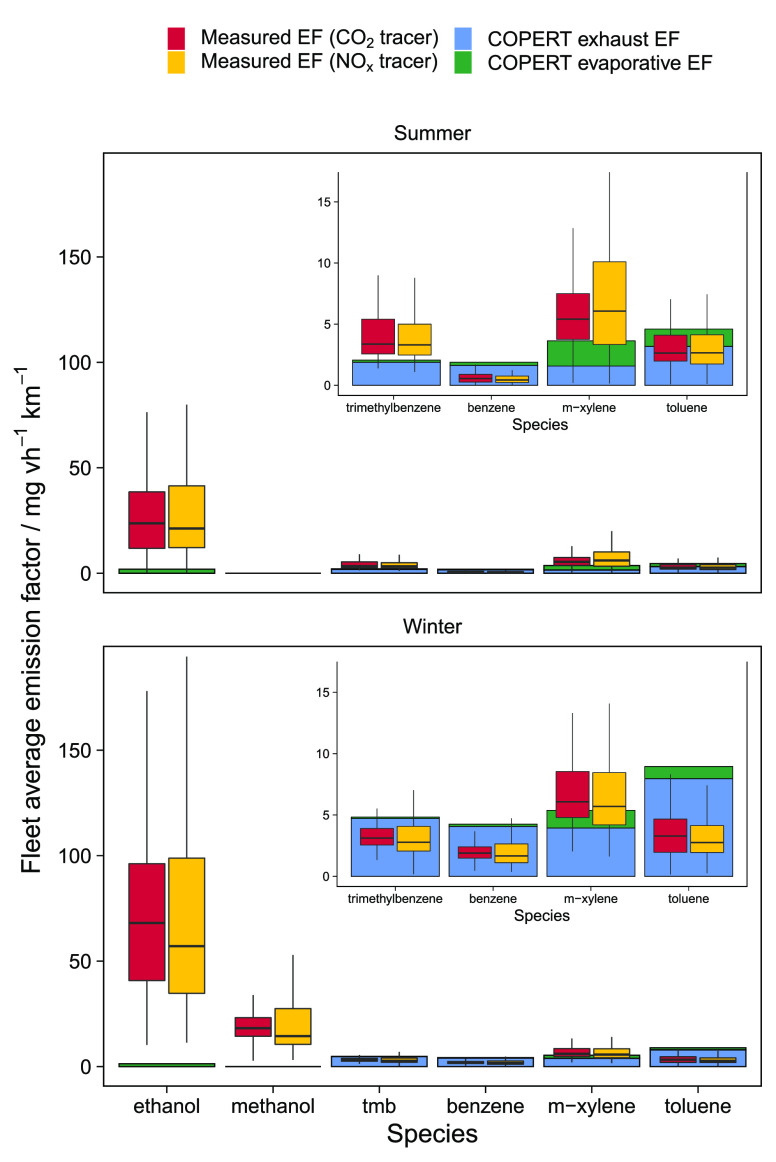
Box plots of measured VOC emission factors at the Manchester roadside using CO_2_ and NO_*x*_ as tracer species, compared to COPERT calculated exhaust and evaporative emission factors as bars, faceted by season. In the top right of each facet is an expanded view of the aromatic emission factors to improve clarity.

Emission factors were calculated using both NO_*x*_ and CO_2_ as the tracer species due to their high degree of emissions regulation and therefore relatively well-known emission factors. Calculations using both enabled a useful comparison to be made as most previous roadside increment-type analyses have used NO_*x*_ only as a tracer.^19,20^ However, the accuracy of NO_*x*_ emission factors in emissions inventories has been questioned as a result of the diesel-gate scandal and the ineffectiveness of exhaust treatment systems under real-world driving conditions, especially in urban areas.^10^ Dilution factors were reasonably consistent with those measured previously in London and can be seen diurnally in Figure S2, with summer dilution factors being 21% and 24% larger than winter for CO_2_ and NO_*x*_ respectively.^10^Figure 4 shows that in general, there is a good level of agreement between the two tracer methods; using NO_*x*_ as a tracer gave VOC emission factors that were on average 5% lower in summer and 16% lower in winter. This is due to a likely underestimation of NO_*x*_ in the emissions used in the calculations with the temperature-related performance of NO_*x*_ emissions control technologies explaining the seasonal variability. For the remainder of this analysis, only the CO_2_ emission factors will be discussed.

The aromatic species had emission factors in the range of 1–6 mg vh^–1^ km^–1^ and were similar across the seasons as a result of the competition between increased fuel evaporative emissions in the summer and increased cold exhaust emissions in the winter. The measured emission factors agreed well with COPERT-derived values with small discrepancies between the species arising due to variability in the speciation of VOC emissions from road transport in the inventory, itself influenced by fuel blends. Winter aromatic emission factors were slightly overestimated in COPERT due to greater cold exhaust emissions, all of which would not be captured at the roadside site. Nevertheless, the general agreement between in-field measured emission and COPERT for aromatics is very encouraging, but perhaps not surprising given the long intensive focus that regulations have had on VOCs of this type and exhaust emissions.

Emission factors for ethanol and methanol were much higher at 68 ± 42 and 18 ± 7 mg vh^–1^ km^–1^, respectively, for winter, and 24 ± 19 and 12 ± 10 mg vh^–1^ km^–1^ for summer. Since a summer methanol emission factor was unavailable from the roadside increment methodology due to the urban background interference, an estimate was calculated from the average speciation of screenwash blends on the market in the U.K. and the summer ethanol emission factor. Since the composition of major sources such as screenwash are not well-known, and variable between products, we conducted laboratory headspace analysis of a range of products to evaluate the relative speciation between ethanol and methanol. This is shown in the SI, indicating that an apportionment of 67:33 ethanol/methanol would be reasonable at this time. This is, within error, in agreement with the relative proportions of the ethanol and methanol emission factors measured for summer in this study. (We note that while methanol is now regulated and limited as an additive in Europe, online retailers appear to sell materials that continue to contain high methanol content.) Assuming the speciation at the measurement site was the same as that measured in the lab and applying it to the ethanol emission factor gave the summer methanol emission factor of 12 ± 10 mg vh^–1^ km^–1^. The emissions of the two alcohol species were substantially underestimated by the COPERT methodology.

The discrepancy can be rationalized and the VOC budget closed by also including an emission of nonfuel, nonexhaust (NFNE) VOC deriving from what are classified as “car care” solvents in the NAEI.^25^ A large fraction of this is thought to be screenwash leading to a release of ∼35 kT of VOCs in 2018. Dividing through by the total number of vehicle kilometers traveled in 2018 in the U.K. (537 billion km, DfT Road Transport Statistics) gives an estimated emission factor based on solvent inventories for screenwash of 64 mg vh^–1^ km^–1^; a figure that agrees remarkably well with the seasonal average of the combined ethanol and methanol median emission factors measured in this study (60 ± 39 mg vh^–1^ km^–1^). The increased winter emission factor is then explained by increased screenwash usage in wetter and dirtier conditions.

### Implications

#### For Future Emissions and Policy

A large source of vehicle emissions not captured by international emissions methodologies such as COPERT is surprising but reflects that the historical focus has been overwhelmingly on fuel-related exhaust and evaporative emissions. It is a measure of the success of abatement technologies that these are now so reduced that other NFNE sources become visible. Real-world observations of NFNE are, in practice, in good agreement with separate industry solvent use statistics, so to a degree have been “hiding in plain sight”. An important feature of car care product emissions is that they are independent of fuel type, meaning the emissions are applicable to all vehicles including those powered by battery electric powertrains. Therefore, we use this information to propose the need for a direct VOC emission factor for electric vehicles in international methodologies that are used to quantify the impacts of road transport on air quality. Subtracting the COPERT-estimated ethanol and methanol emissions (arising from exhaust and evaporative losses of ethanol-blended fuel use in the U.K.) from the measured sum of the alcohol emission factors gives the NFNE-related emission factor. We propose a value of 58 ± 39 mg vh^–1^ km^–1^ for U.K. vehicles which assumes all emissions can be apportioned as a combination of ethanol and methanol with an apportionment of 67:33 ethanol/methanol as derived from the laboratory headspace analysis of screenwash blends.

**Figure 5:**
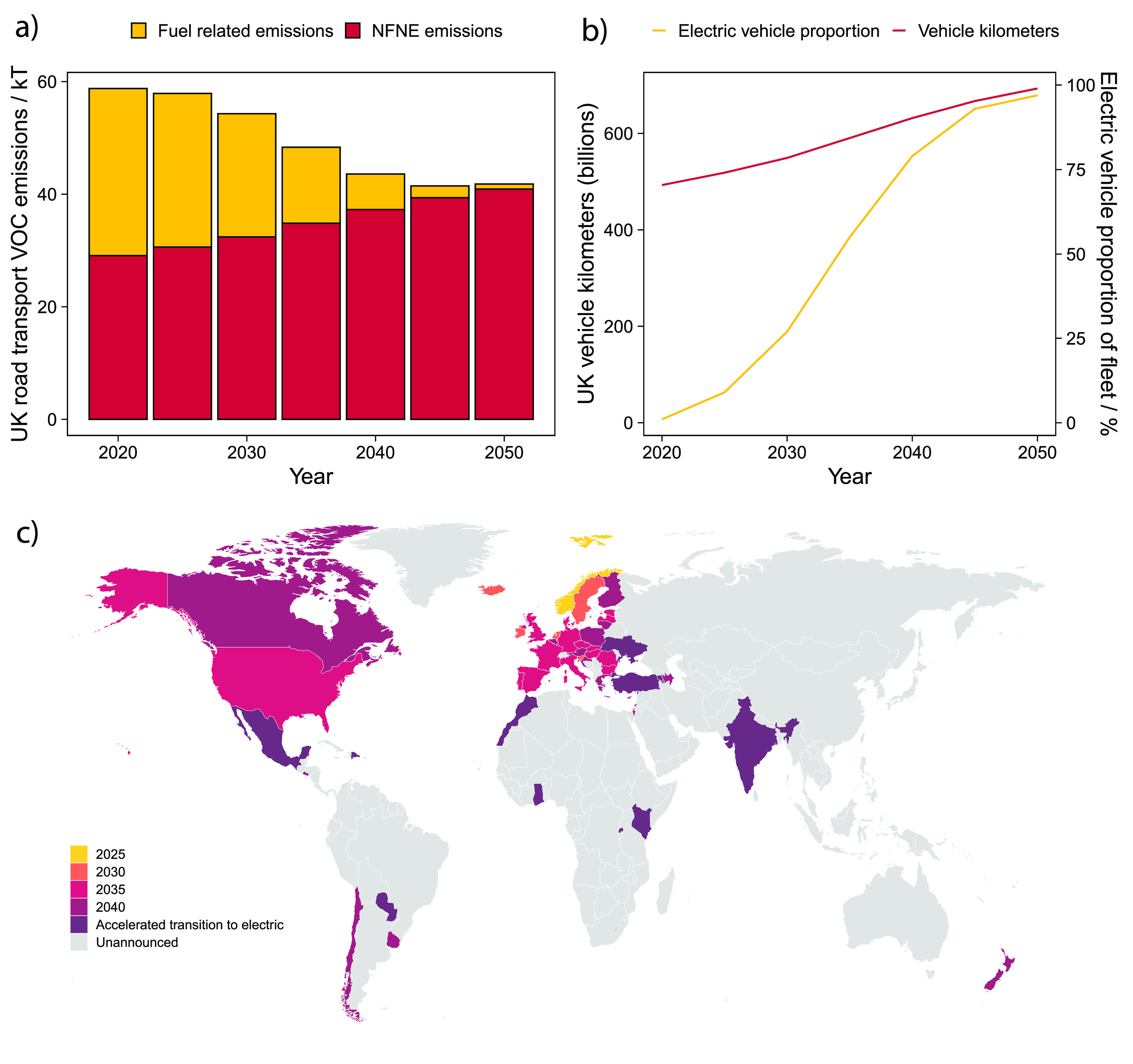
(a) VOC emission projections for road transport for fuel-related and NFNE sources. Fuel-related emissions are proportionally reduced from 2020 estimates using projected electric vehicle fleet percentage. NFNE emissions are generated by multiplying the derived emission factor in this work (58 mg vh^–1^ km^–1^) by projected vehicle kilometers traveled in the U.K. (b) Electric vehicle proportion and UK annual vehicle kilometers traveled predictions used to produce (a) from DfT (Road Traffic Forecasts 2018). (c) A global timeline of commitments to the banning of new petrol and diesel vehicle sales. Data were mainly taken from COP 26 signatory list, but also from the EU “Fit for 55” proposal and the US Executive Order on Catalyzing Clean Energy Industries and Jobs through Federal Sustainability.

A NFNE emission factor of 58 mg vh^–1^ km^–1^ is 1.8 x greater than total exhaust VOC emissions in the U.K. (32 mg vh^–1^ km^–1^). Looking to the future, NFNE emissions from solvent products may actually increase, should overall vehicle mileage increase, as is indicated in some projections of future electrified transport fleets. Annual vehicle kilometers driven in the U.K. have steadily increased over the last three decades and are predicted to increase by up to 51% on 2015 levels by 2050.^26^ This is a consequence of increased population and an anticipated reduction in the cost of travel in electric vehicles. Assuming that NFNE emissions are simply proportional to total vehicle distance traveled, and that fuel-related emissions are inversely proportional to the % of electric vehicles in the U.K. fleet, future road transport emissions can be estimated. Figure 5 shows this projection; because of fleet electrification there is initially a fall in total road transport VOC emissions but due to increasing vehicle km and the related scaling in NFNE emissions, road transport VOC emissions begin to increase after 2045. NFNE emissions would represent ∼40 kT of VOC emissions in 2050 which is around 6% of the U.K.’s 2030 National Emission Ceilings Directive (NECD) ceiling. Although only U.K. figures are presented here, this is a transition of global significance. The main driver for the rate of fleet electrification is transport decarbonization and the policy to ban new sales of petrol and diesel vehicles. Current ICE ban commitments are highlighted in Figure 5 and span all the major continents, with further pledges, particularly in Asia, expected soon.

#### For Atmospheric Chemistry

The transition from fuel-related VOC emissions to nonfuel-related VOC emissions represents a notable change in the VOC speciation of the emissions. Fuel-related VOC emissions contain a complex mixture of aliphatics and aromatics whereas NFNE emissions typically only contain ethanol and methanol. On average, the ozone formation potential of ethanol and methanol is lower than a similar overall mass emission of fuel related VOCs.^27^ However, increasing concentrations of ethanol in the atmosphere and even screenwash-related methanol emissions have been associated with increased formation of tropospheric ozone.^28,29^ A useful comparison can be drawn with bioethanol use in Brazil. Ozone and PM levels have been shown to increase during periods where more ethanol is combusted in vehicles compared to gasoline, despite ethanol combustion typically reducing VOC emissions.^30,31^ Here, the cause is thought to be reduced NO_*x*_ emissions in a VOC limited ozone regime. During fleet electrification, a similar scenario could occur in which VOC emissions remain high as a result of NFNE emissions with reductions in NO_*x*_ increasing ozone and PM formation. While the transition could, at least initially, reduce the urban VOC burden, models which do not include NFNE emissions may underestimate future urban ozone concentrations due to the size of the missing source.

In addition to its role in ozone formation, ethanol is a key precursor to the formation of acetaldehyde which is a highly reactive compound that is also a suspected carcinogen and is associated with various respiratory conditions.^32^ Increasing ethanol concentrations in the atmosphere have been associated with increased production of peroxyacetyl nitrate (PAN) under high NO_*x*_ conditions.^28^ PAN is an important species for the atmospheric transport of NO_*x*_ with implications for the global distributions of ozone and OH.^33^ However, there is uncertainty surrounding future emissions scenarios of NO_*x*_ due to a poor quantification of the impact of congestion and the proposed transitions from natural gas to hydrogen combustion in heating systems.^34,35^ As such, the ramifications of increasing ethanol concentrations are currently unknown but potentially important. Methanol plays an important atmospheric role through involvement in hydroxyl radical cycling and thus the tropospheric oxidative capacity.^36^ It is also a precursor for formaldehyde and CO.

Looking forward, we recommend the inclusion of NFNE emissions within road transport emissions methodologies and within the COPERT framework. Road transport activity statistics are much more commonly reported worldwide than industrial sales of screenwash. This emission factor in a per kilometer form makes the calculation accessible for all. Moreover, road transport is a unique VOC source due to the coemission of NO_*x*_ and the emission location largely occurring in heavily populated areas. Assignment of emissions via COPERT and vehicle mileage geolocates the VOC emissions where they actually occur, whereas the spatial desegregation of emissions of VOC in the industrial solvents class can often be represented as a uniform emission. This may help improve the performance of local to regional air pollution models. With bans on the sale of petrol and diesel vehicles starting as early as 2025 in some countries, it is crucial the atmospheric impacts of this transition are properly represented and monitored. For this, there is a clear benefit to having emissions associated with the correct sector. It is possible, however, that the NFNE emission could be relatively straightforwardly reduced through policies (or voluntary schemes) that required product reformulation to remove VOCs of air pollution significance. Current approaches to VOC emissions control under EMEP and CLRTAP do not discriminate by VOC reactivity or photochemical ozone creation potential. While this may not necessarily appear to be an optimal regulatory response, control of methanol and ethanol could be a potentially effective mechanism for a country to reduce overall mass emissions in response to further lowering of emission ceilings.

## Supporting Information Available

The following files are available free of charge. The Supporting Information is available free of charge at https://pubs.acs.org/doi/10.1021/acs.est.3c00845.

Figure S1, measurement locations; Figure S2, dilution factor diurnal profiles; Table S1, SIFT-MS measured ions; Table S2, screenwash headspace analysis results; Table S3, measured summer VOC emission factors; Table S4, measured winter VOC emission factors; and Table S5, COPERT emission factors (PDF)
