# Supplementary material for: Unreported VOC Emissions from Road Transport Including from Electric Vehicles
Source: Environ Sci Technol. 2023 May 16;57(21):8026–34. doi: 10.1021/acs.est.3c00845 (PMC10233797; doi:10.1021/acs.est.3c00845)
Supplement: Supplementary file 1 — es3c00845_si_001.pdf [file es3c00845_si_001.pdf]

Unreported VOC emissions from road transport including from electric vehicles

Samuel J. Cliff,[*,†] Alastair C. Lewis,[†] Marvin D. Shaw,[†] James D. Lee,[†] Michael Flynn,[‡] Stephen J. Andrews,[†] James R. Hopkins,[†] Ruth M. Purvis,[†] and Amber M. Yeoman[†]

†*Wolfson Atmospheric Chemistry Laboratories, University of York, York, YO10 5DQ, UK*

‡*School of Earth and Environmental Sciences, University of Manchester, Manchester, M13 9PL, UK*

E-mail: samcliff1@googlemail.com

Supporting Information

This SI contains 10 pages of information containing 2 figures and 5 tables.

GC conditions

Air was drawn down a 2 m 1/4" Silonite (Entech Instruments Inc., USA) coated heated stainless steel tube at 20-30 slpm using a MB-158 metal bellows compressor (Senior Aerospace BWT., UK) and back pressure regulated to maintain a 25 sccm sampling flow through a custom built pre-concentration unit. Calibration gas was provided from a working standard cylinder comprising a sample of NMVOCs (material number: 177664-AL-HC, BOC Special Gases) diluted to 1.2 ppb per component in purified nitrogen (cylinder number: D035781, Air Liquide S.A., France), linked to an NPL30 primary calibration standard (National Physical Laboratory, UK). VOC free blanks were created from compressed air, through a bed of 1 % Pt on Alumina beads at 375 °C. Air, calibration and blanks were de-humidified with a -40 °C water trap and 500 mL samples were pre-concentrated on a multi-bed sorbent trap at around -120 °C, flow controlled by a downstream MC mass flow controller (Alicat Scientific Inc., USA). After removal of CO_2 and other permanent gases at -80 °C, the preconcentration trap was desorbed and refocused before final desorption in a H_2 carrier gas flow onto a VF-WAX-MS GC column (60 m, 0.25 mm I.D. 150 μm film, Agilent Technologies Inc., USA). Analytes not retained on the WAX column (C2-C6 aliphatics) were diverted by Deans Switch and separated on a PLOT column ($A1_2O_3/Na_2SO_4$ 50 m, 0.32 mm I.D. 5 μm film, Agilent Technologies Inc., USA). Oxygenates, > C6 aliphatic, and aromatics were not diverted and analytes eluting from each column were detected by flame ionisation detectors.

Screenwash composition

The alcohol composition of 10 different screenwash products ordered online (available UK market, October 2022) were determined by gas chromatography time of flight mass spectrometry. A headspace analysis was conducted, with the results shown in Table S2.

COPERT Calculations

To calculate emissions in COPERT, various data inputs are required. These were taken from the Ricardo handbook, or, if unavailable or Manchester specific, sourced elsewhere.[1] The data used is described below.

- Year: 2021 was used for the summer experiments and 2022 was used for the winter experiments.
- Environmental information (min temperature, max temperature and humidity): obtained from weather data archives for the different measurement periods. All months are filled with the same values so that the annual emissions represent conditions during the desired month. Summer: 12 °C, 20 °C and 80 %. Winter: 2 °C, 8 °C and 87 %.
- Trip length: 10 km, as described in the Ricardo handbook.
- Fuel advanced specifications = 2009 fuel year (the most recent).
- Reid Vapour Pressure = Summer: 70 kPa, Winter: 90 kPa, as described in the Ricardo handbook.

Stock configuration and activity data was obtained from the nearby traffic camera but required some additional processing for input into COPERT. COPERT requires a high level of granularity in the stock configuration and activity input. Vehicles should be broken down into the various vehicle technologies by vehicle category (e.g. passenger car, HGV, bus etc.), Euro Standard, fuel type and size. Unfortunately, the traffic camera data available to us only breaks down the traffic into vehicle category. In order to further break this down into the required granularity, each vehicle category counts was multiplied by the 2021 or 2022 corresponding UK average Euro Standard, fuel type and vehicle size fractions taken from the NAEI fleet composition projections (2019),[2] as shown in Eq. 1.

$$n_{c,e,f,s} = n_c \times e_c \times f_c \times s_c \tag{1}$$

Where:

- $n_{c,e,f,s}$ is the number of vehicles of category c, Euro Standard e, fuel type f and size s.
- n_c is the number of vehicles of category c, measured at the roadside site
- e_c is the fraction of vehicles of category c that are of Euro Standard e
- f_c is the fraction of vehicles of category c that are of fuel type f
- s_c is the fraction of vehicles of category c that are of size s

Here, the assumption that the traffic measured in Manchester is representative of the whole UK fleet is made. In addition to fleet breakdown and vehicle counts, mean annual activity and lifetime cumulative mileage is required. Mean annual activity for the UK is $\sim$ 12000 km and the average age of a car is $\sim$ 8.4 year old. Therefore, lifetime cumulative mileage is given as 100000 km (the product of the two). Lifetime cumulative activity is largely required for degradation parameters, and it was found that it has minimal impact on the emissions outputted by COPERT. Mean activity is used for the calculation of annual bulk emissions. However, the actual magnitude is not important as we divide through by it later during conversion to an EF. Finally, circulation activity was divided into a 100 % share for urban driving to represent our Manchester site, using urban peak/off peak traffic speeds obtained from the nearby traffic camera ($\sim$ 40/50 km h^{-1}).

All other data was kept as standard in the COPERT database. This includes fuel evaporation data such as fuel tank size, carbon canister size and % fuel injection. In the NAEI handbook, it is assumed "that all pre-Euro 1 cars would be with carburettor and that all Euro 1 onward cars would use fuel injection, but with fuel return systems, hence, having high emission factors". There is no input for % fuel return/returnless fuel systems in this version of COPERT so this impact can not be studied here.

Conversion to emission factors

COPERT outputs hot, cold and evaporative bulk NMVOC emissions for each vehicle technology. Evaporative emissions consist of a number of sources that we do not want to include in this analysis. As mentioned earlier, diurnal and hot soak emissions occur when the engine is turned off and so are not present at the roadside site. Therefore, rather than use the bulk evaporative emissions, the EFs in COPERT used for running emissions calculation are used instead. A fleet average running evaporative emissions factor is calculated using Eq. 2:

$$EF_{r,fa} = \frac{\sum_x EF_{r,x} \times n_x}{n_{total} \times 10\ km} \tag{2}$$

Where:

- $EF_{r,fa}$ is the fleet average running emissions factor in g vh^{-1} km^{-1}
- $EF_{r,x}$ is the running emissions factor for vehicles of technology x in g trip^{-1}
- n_x is the number of vehicles of technology x
- n_{total} is the total number of vehicles measured
- 10 km is the average trip length and thus division by it converts the emissions factor from g trip^{-1} to g km^{-1}.

Exhaust emissions are calculated using Eq. 3:

$$EF_{e,fa} = \frac{E_{a,hot} + E_{a,cold}}{\sum_x n_x \times d_{a,x}} \tag{3}$$

Where:

- $EF_{e,fa}$ is the fleet average exhaust emission factor in g vh^{-1} km^{-1}
- $E_{a,hot}$ is the annual hot NMVOC emissions in g
- $E_{a,cold}$ is the annual cold NMVOC emissions in g

- n_x is the number of vehicles of technology x
- $d_{a,x}$ is the annual distance travelled per vehicle of technology x in km

The evaporative and exhaust NMVOC EFs are separately speciated based on their corresponding speciation fractions in COPERT, also displayed in the EMEP/EEA guidebook, in Eq. 4:

$$EF_{x,i} = EF_i \times s_x \tag{4}$$

Where:

- $EF_{x,i}$ is the emission factor for species x of emission type i (exhaust or evaporative) in g vh^{-1} km^{-1}
- EF_i is the emission factor for emission type i in g vh^{-1} km^{-1}
- $s_{x,i}$ is the percentage fraction of species x in emissions of type i

It should be noted that in COPERT ethanol is not included as a fraction in either exhaust or evaporative emissions. However, it can be calculated as the remaining fraction once all other species have been subtracted from 100 %. The 6 % given for evaporative emissions and 0 % given for exhaust emissions is consistent with that given in the EMISIA/EEA handbook.

Finally, the total EF for a NMVOC species is calculated from the sum of the speciated exhaust and evaporative EFs in Eq. 5:

$$EF_x = \sum_i EF_{x,i} \tag{5}$$

Where:

- EF_x is the emission factor for species x in g vh^{-1} km^{-1}
- $EF_{x,i}$ is as above

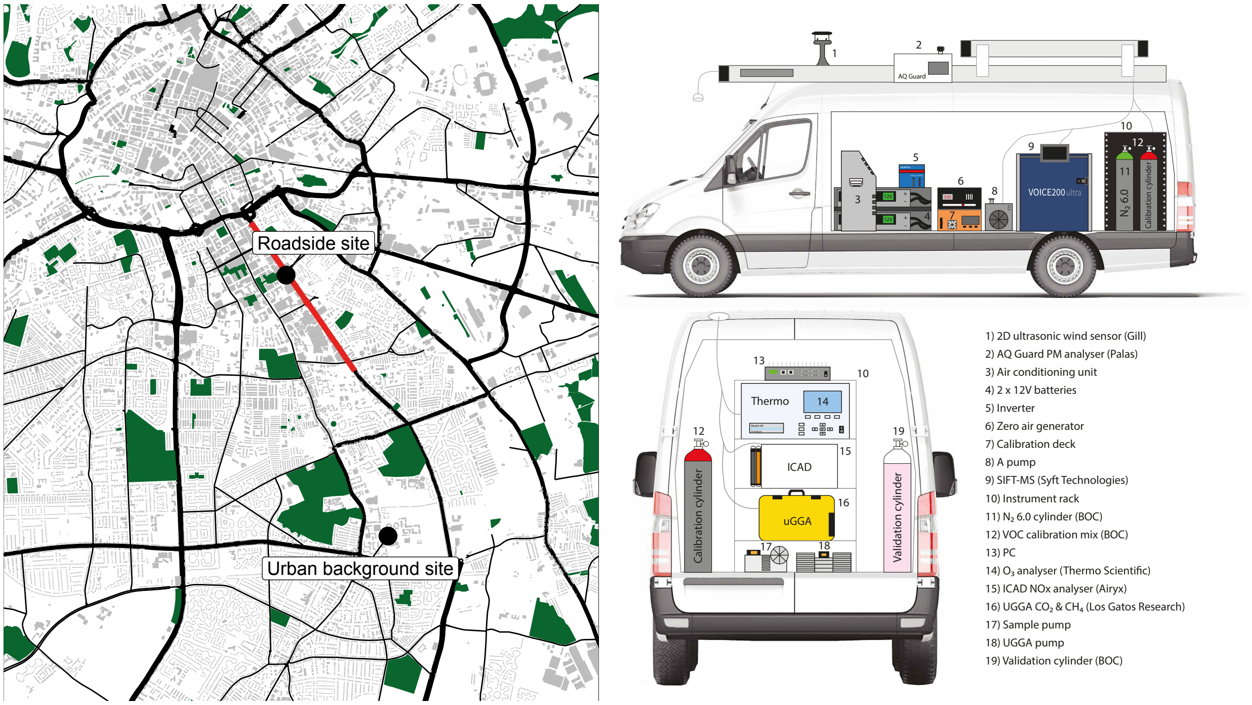

Figure S1: Left: Locations of the roadside and urban background measurement sites (black dots) and Upper Brook Street (red line) in Manchester, UK. Map reproduced with permission in R using data from © OpenStreetMap contributors, available under the Open Database License. Right: A schematic of the WASP instrumentation configuration during OSCA. At the top is a side profile with each piece of kit numbered and labeled below. Below is a back profile of the black instrument rack **10**.

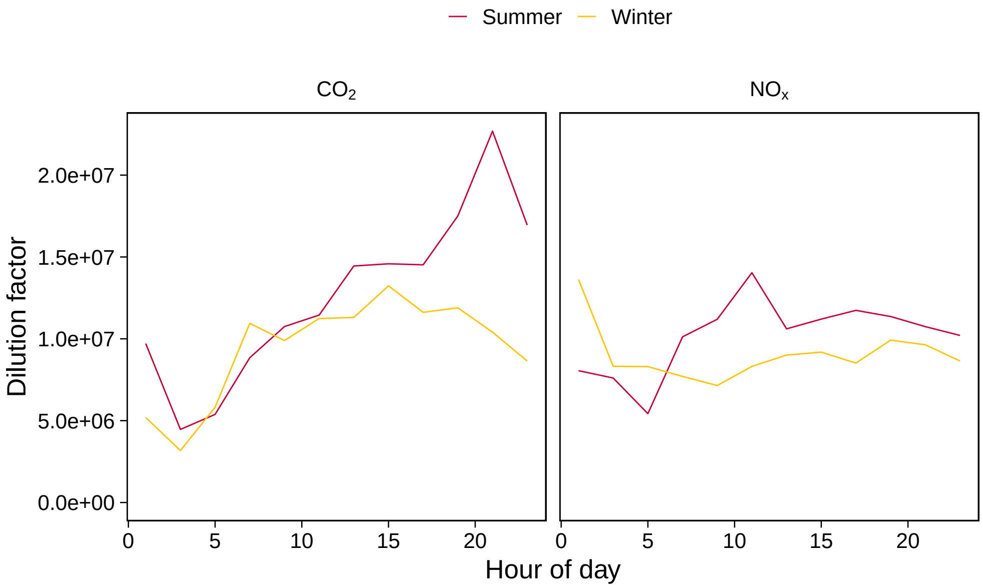

Figure S2: Diurnal profiles of the dilution factors measured for NO_x and CO_2 during the summer and winter measurement periods.

Table S1: A list of the compounds measured by the SIFT-MS during the OSCA experiments, their corresponding product ion and molar mass, a flag to indicate the calibration status, all grouped by the reagent ion used.

Reagent Ion	Compound	Product ion	Molar Mass g mol^{-1}	Calibrated?
H_3O^+	Methanol	CH_5O^+	33	Y
	Ethanol	$C_2H_7O^+$	47	Y
	Acetonitrile	$CH_3CN{\cdot}H^+$	42	Y
	Acetaldehyde	$C_2H_4O{\cdot}H^+$	45	N
	Nitrous acid	$H_2NO_2{}^+$	48	N
	Nonane	$C_9H_2O{\cdot}H_3O^+$	147	Y
	Decane	$C_{10}H_{22}{\cdot}H_3O^+$	161	Y
	Formaldehyde	CH_3O^+	31	N
NO^+	Benzene	$C_6H_6{}^+$	78	Y
	Toluene	$C_7H_8{}^+$	92	Y
	M-xylene	$C_8H_{10}{}^+$	106	Y
	1,2,4-trimethlybenzene	$C_9H_{12}{}^+$	120	Y
	Acetone	$C_3H_6O{\cdot}NO^+$	88	Y
	Butadiene	$C_4H_6{}^+$	54	Y
	Isoprene	$C_5H_8{}^+$	68	Y
	3-buten-2-one	$C_4H_6O{\cdot}NO^+$	100	Y
	Butanone	$C_4H_8O{\cdot}NO^+$	102	Y
	Acetic acid	$CH_3COOH{\cdot}NO^+$	90	N
	Methyl tert-butyl ether	$C_4H_9{}^+$	57	N
	Octane	$C_8H_{17}{}^+$	113	Y
$O_2{}^+$	Isoprene	$C_5H_7{}^+$	67	Y
	Acetylene	$C_2H_2{}^+$	26	N
	Propane	$C_2H_4{}^+$	28	N
	Nitrogen dioxide	$NO_2{}^+$	46	N

Table S2: The headspace composition of 10 different screenwash products as determined in the laboratory.

Product	Methanol %	Ethanol %
1	25.1	3.41
2*	4.95	17.5
3*	4.54	7.75
4	4.53	0.00
5	4.50	8.12
6	4.11	18.3
7	0.00	0.00
8*	1.95	7.29
9*	4.58	22.8
10	0.00	22.5
Average	5.42	10.8

*Diluted, ‘ready to use‘ blends

Table S3: Measured summer VOC emission factors calculated using CO_2 and NO_x as tracer species, listed as median and upper and lower quartiles.

Species	Measured emission factor (mg vh^{-1} km^{-1})					
	CO_2			NO_x		
	median	Q1	Q3	median	Q1	Q3
trimethlybenzene	3.367	2.564	5.401	3.307	2.471	5.002
benzene	0.5527	0.2633	0.8936	0.4344	0.2282	0.7525
ethanol	23.69	11.82	38.57	21.24	12.14	41.45
m-xylene	5.406	3.759	7.500	6.069	3.335	10.10
toluene	2.636	1.979	4.096	2.653	1.740	4.129

Table S4: Measured winter VOC emission factors calculated using CO_2 and NO_x as tracer species, listed as median and upper and lower quartiles.

Species	Measured emission factor (mg vh^{-1} km^{-1})					
	CO_2			NO_x		
	median	Q1	Q3	median	Q1	Q3
1,2,4-trimethlybenzene	3.121	2.552	3.899	2.780	2.060	4.077
benzene	1.895	1.483	2.402	1.663	1.112	2.642
ethanol	68.08	40.78	96.19	57.11	34.75	101.7
m-xylene	6.075	4.792	8.543	5.695	4.195	8.451
methanol	18.22	14.37	23.21	14.45	10.55	27.51
toluene	3.287	1.959	4.667	2.755	1.946	4.139

Table S5: COPERT calculated total, exhaust and evaporative emission factors for the studied VOC species, separated by season.

Species	COPERT emission factor / mg vh^{-1} km^{-1}					
	Summer			Winter		
	Total	Exhaust	Evaporative	Total	Exhaust	Evaporative
trimethlybenzene	2.059	1.882	0.1771	4.831	4.708	0.1233
benzene	1.884	1.627	0.2576	4.249	4.069	0.1794
ethanol	1.932	0.000	1.932	1.345	0.000	1.345
m-xylene	3.635	1.574	2.061	5.374	3.939	1.435
methanol	0.000	0.000	0.000	0.000	0.000	0.000
toluene	4.601	3.184	1.417	8.951	7.964	0.9866

References

(1) Ricardo Energy & Environment. Methodology for the UK's Road Transport Emissions Inventory, version for the 2016 National Atmospheric Emissions Inventory. https://naei.beis.gov.uk/reports/reports?report_id=957 (accessed 2022-11-21).

(2) National Atmospheric Emissions Inventory 2019. Defra and BEIS, licenced under the Open Government Licence (OGL), Crown Copyright 2020; naei.beis.gov.uk/data/.
